# Distribution of Cardiac and Renal Corin and Proprotein Convertase Subtilisin/Kexin-6 in the Experimental Model of Cardio-Renal Syndrome of Various Severities

**DOI:** 10.3389/fphys.2021.673497

**Published:** 2021-10-18

**Authors:** Emad E. Khoury, Ahmad Fokra, Safa Kinaneh, Yara Knaney, Doron Aronson, Zaid Abassi

**Affiliations:** ^1^Department of Physiology, Bruce Rappaport Faculty of Medicine, Technion–Israel Institute of Technology, Haifa, Israel; ^2^Department of Cardiology, Rambam Health Care Campus, Haifa, Israel; ^3^Department of Laboratory Medicine, Rambam Health Care Campus, Haifa, Israel

**Keywords:** corin, PCSK6, natriuretic peptides, furin, cardiorenal syndrome, heart, kidney

## Abstract

Congestive heart failure (CHF) often leads to progressive cardiac hypertrophy and salt/water retention. However, its pathogenesis remains largely unclarified. Corin, a cardiac serine protease, is responsible for converting proANP and proBNP to biologically active peptides. Although the involvement of corin in cardiac hypertrophy and heart failure was extensively studied, the alterations in corin and proprotein convertase subtilisin/kexin-6 (PCSK6), a key enzyme in the conversion of procorin to corin, has not been studied simultaneously in the cardiac and renal tissues in cardiorenal syndrome. Thus, this study aims to examine the status of PCSK6/corin in the cardiac and renal tissues of rats with CHF induced by the creation of aorto-caval fistula (ACF). We divided rats with ACF into two subgroups based on the pattern of their urinary sodium excretion, namely, compensated and decompensated. Placement of ACF led to cardiac hypertrophy, pulmonary congestion, and renal dysfunction, which were more profound in the decompensated subgroup. Corin immunoreactive peptides were detected in all heart chambers at the myocyte membranal and cytosolic localization and in the renal tissue, especially in the apical membrane of the proximal tubule, mTAL, and the collecting duct. Interestingly, the expression and abundance of corin in both the cardiac ventricles and renal tissues were significantly increased in compensated animals as compared with the decompensated state. Noteworthy, the abundance of PCSK6 in these tissues followed a similar pattern as corin. In contrast, furin expression was upregulated in the cardiac and renal tissues in correlation with CHF severity. We hypothesize that the obtained upregulation of cardiac and renal PCSK6/corin in rats with compensated CHF may represent a compensatory response aiming at maintaining normal Na^+^ balance, whereas the decline in these two enzymes may contribute to the pathogenesis of avid sodium retention, cardiac hypertrophy, and blunted atrial natriuretic peptide/brain natriuretic peptide actions in decompensated CHF.

## Introduction

Heart failure (HF) is a clinical syndrome characterized by inadequate peripheral blood flow, leading to the suffering of additional vital organs. In this context, the kidney is an important player, as poor renal blood supply in HF leads to a deleterious clinical setting named cardiorenal syndrome (CRS), which develops in approximately half of the HF patients (Damman and Testani, 2015; Mazurek and Jessup, 2017).

Cardiorenal syndrome involves multiple interdependent mechanisms, including the activation of neurohormonal factors, such as renin-angiotensin-aldosterone system and sympathetic nervous system, and also the increased secretion of endothelin-1, and antidiuretic hormone (ADH) (Schefold et al., 2016). Persistent activation of the neurohormonal factors results in deleterious outcomes, including sodium and water retention, along with decreased free water clearance, renal and systemic vasoconstriction, and also cardiac and renal tissue remodeling (Mazurek and Jessup, 2017). On the other hand, the natriuretic peptides (NPs) system plays a key role in opposing the abovementioned detrimental systems. *Via* its two cardiac natriuretic hormones, atrial natriuretic peptide (ANP) and brain natriuretic peptide (BNP), the NPs system promotes natriuresis, diuresis, decreases sodium reabsorption, and diminishes ADH secretion (Goetze et al., 2020). In addition, the activation of the NPs system results in vasodilation and lower blood pressure, reduced renal sympathetic activity, and attenuation of cardiac, renal, and vascular tissue remodeling (Goetze et al., 2020).

One of the contradictions seen in HF and CRS is the supremacy of the deleterious neurohormonal systems over the beneficial NPs, despite the remarkably elevated circulating levels of ANP and BNP (Villarreal and Freeman, 1991; Braunwald, 2008; Chaney and Shaw, 2010; Dries, 2011; McMurray et al., 2012; Yancy et al., 2013; Schefold et al., 2016). Several mechanisms have been suggested to explain this phenomenon, yet the involvement of the NPs machinery system has not been studied thoroughly (Charloux et al., 2003; Bouley, 2012; Ngo et al., 2013; Egom et al., 2015). Corin, a type-II transmembrane serine protease found mainly in cardiomyocytes, is essential for converting the proANP and proBNP hormones into their active forms (Yan et al., 1999, 2000; Wu et al., 2002; Semenov et al., 2010; Ichiki et al., 2011). To gain biological activity, corin must first undergo enzymatic cleavage by proprotein convertase subtilisin/kexin-6 (PCSK6, also named PACE4) to be activated (Seidah et al., 2013; Chen et al., 2015). In addition, corin is present in the circulation due to metalloprotease ADAM10-mediated shedding and autocleavage (Wang et al., 2015).

Although some studies have found that cardiac corin undergoes downregulation in HF, other studies reported opposite findings (Langenickel et al., 2004; Tran et al., 2004; Jiang et al., 2005; Calderone et al., 2006; Chen et al., 2010; Gladysheva et al., 2013; Ichiki et al., 2013). These conflicting observations may stem from several factors, including the application of different HF models with distinct duration and severity of the disease, inconsistency in the cardiac chamber being investigated, and the use of different analytical methodologies across these studies. Moreover, the status of renal corin has not been studied in HF so far. The kidney is an important site for the local generation of NPs, where corin is expressed at various sites of the nephron (Dong et al., 2016). The renal corin and *in situ* NPs assumedly play an essential role in the local production of ANP, thus contributing to water and salt balance under normal and disease states, including congestive heart failure (CHF). Finally, PCSK6, a recently discovered enzyme and of paramount importance for the normal activity of corin (Chen et al., 2015), has not been studied in HF before.

Therefore, the present study was designed to examine the expression of corin and PCSK6 in the cardiac and renal tissues, simultaneously, and the subsequent changes in the NPs (ANP and BNP) in an experimental model of HF of different severities, induced by the placement of aorto-caval fistula (ACF). Most recently (Khoury et al., 2018), we utilized this experimental model to study the status of PCSK6/corin in the pulmonary tissue of rats with HF. Exploring the alterations in cardiac and renal PCSK6/corin/NPs axis in HF of distinct severities is appealing, as it may provide new insights into the pathogenesis of cardiac remodeling and renal dysfunction characterizing this clinical setting.

## Materials and Methods

The samples used in the current study were collected from the same animals utilized in our animal CHF rat model as previously described (Khoury et al., 2018). While our former study focused on the pulmonary tissue, the current research analyzes the cardiac and renal specimens. Briefly, studies were performed on male Sprague–Dawley rats (Harlan Laboratories, Jerusalem, Israel), weighing 300–350 g. The rats were housed in individual metabolic cages in a temperature-controlled room and fed a standard rodent diet and tap water *ad libitum.* Urinary volume and urinary sodium concentration were measured throughout the entire study period (beginning 4 days prior to surgery). The studies involving animals were reviewed and approved by the Technion Committee for Care and Use of Laboratory Animals.

### Experimental Model

An ACF was surgically created between the abdominal aorta and the inferior vena cava, as reported in detail from our laboratory (Winaver et al., 1988; Abassi et al., 1990, 2001). Briefly, under sodium pentobarbitone anesthesia (60 mg/kg BW, i.p.), the abdominal aorta and inferior vena-cava were exposed through a mid-abdominal incision. The outer wall of the vena-cava was opened, and a fistula (∼1.2 mm outer diameter) was surgically created in the common wall between the vessels. The opening in the wall of the vena cava was closed with a continuous suture. A matched group of sham-operated rats that underwent laparotomy only served as controls. The animals were allowed to recover and then returned to their metabolic cages for monitoring urine output and sodium excretion for an additional 7 days. Based on their urinary sodium excretion (UNaV), rats with ACF were divided into two subgroups: compensated (UNaV > 1200 μEq/day) and decompensated (UNaV < 200 μEq/day), accompanied by evidence of fluid retention (Winaver et al., 1988). Animals from the various groups (*n* = 7–8) were anesthetized, tracheotomized, and polyethylene catheters (PE50) were inserted into the carotid artery and jugular vein.

### Cardiac Function

Cardiac function was monitored by inserting a Millar cardiac conductance catheter (Mikro-Tip^®^, Millar Instruments, TX, United States) to the left ventricle (LV) *via* the carotid artery. The system simultaneously measures high-fidelity ventricular pressure, volume, and intracardiac electrocardiography (ECG) *via* a single catheter. The LV pressure and volume signals were plotted against each other in real time, generating the characteristic left ventricular pressure-volume (PV) loops. A stable interval of 1 min was chosen for each rat. The Millar system provided the following measurements along with the raw data (pressure and volume vs. time): stroke volume, cardiac output, heart rate, end-diastolic volume, end-systolic volume, ejection fraction, maximal and minimal ventricular dP/dT.

At the end of the experiments, the heart and the kidneys were washed *via* left ventricle perfusion with 120 ml phosphate-buffered saline (0.01M PBS, pH 7.4) containing heparin (5 U/ml). Then, the heart and the kidneys were harvested and weighed. Cardiac/body weight ratio (HW/BW%), an index for cardiac hypertrophy, was calculated in the various groups of animals. Similarly, the kidney weight to body weight ratio (KW/BW%) was calculated. The collected tissues were subject to western blot and real-time qPCR analysis.

### Heart and Kidney Fixation

Additional groups of rats with compensated and decompensated CHF, and also sham controls (*n* = 3) were anesthetized (Nembotal, 60 mg/kg, i.p.), and their heart and kidneys were fixed *via* carotid artery perfusion, first with 40 ml phosphate-buffered saline (0.02M PBS, pH 7.4) containing heparin (5 U/ml), and then with 220 ml of ice-cold 4% paraformaldehyde in 0.1 M PBS, pH 7.5 containing 4% sucrose. Hearts and kidneys from the different experimental groups were removed and embedded in 10% neutral-buffered formalin. Cardiac and renal tissues samples were then progressively dehydrated in graduated alcohol concentrations (70–100%) and embedded in paraffin.

### Cardiac and Renal Fibrosis

About 5 μm-thick paraffin sections of the cardiac and renal tissues were deparaffinized and rehydrated gradually through 100–70% alcohol solution. Sections were then refixed with Bouin’s solution for 1 h at 56°C followed by staining with Weigert’s iron hematoxylin working solution for 10 min. Tissues were then stained in Biebrich scarlet-acid fuchsin solution followed by phosphomolybdic–phosphotungstic acid solution. Sections were then transferred to aniline blue solution and stained for 10 min. The 1% acetic acid solution was used, followed by dehydration of the sections through 95% ethyl alcohol and absolute ethyl alcohol. Finally, sections were cleared with xylene. All the images were obtained with a computerized scanner.

### Immunofluorescence

Five-micrometer-thick paraffin sections of the various tissues were deparaffinized and rehydrated. Then, slides were subjected to antigen retrieval by using Proteinase K (ab64220, Abcam) for 5 min. Slides were then incubated with 5% normal donkey serum (NDS) in phosphate-buffered saline (PBS) containing 0.3% Tween-20 for 60 min to block non-specific binding and incubated overnight at 4°C with primary antibodies diluted in blocking solution and directed against Corin (1:100, rabbit, ab230311, Abcam) and PCSK6 (1:100, rabbit, ab140934, Abcam). Cy^TM^3 Donkey Anti-Rabbit IgG was used as the secondary antibody (Jackson Laboratories, PA, United States) together with DAPI Fluoromount-G^®^ for nuclear staining. Images were captured using a Widefield Zeiss Upright microscope and analyzed with Zen software. Representative images of the cardiac and renal tissues were obtained at ×40 magnification.

### Western Blot Analysis

Cardiac, renal (both cortical and medullary), and pulmonary tissue samples from the various experimental groups were homogenized on ice. The homogenized tissue was then lysed in RIPA buffer (150 mM NaCl, 1% NP40, 50 mM Tris pH 8.0, 0.5% sodium deoxycholate, and 0.1% SDS) supplemented with a cocktail of protease inhibitors (Roche) in rotation at 4°C for 20 min and then centrifuged at 4°C for 15 min at 12,000 RPM. The cleared supernatant was collected, and the protein concentration was determined (Bradford reagent, Sigma). Equal amounts of extracted proteins (40–60 μg) were loaded and run by electrophoresis on a 9 or 15% SDS–polyacrylamide gel and were transferred to a nitrocellulose membrane. The membranes were incubated in blocking buffer, TBS-T (Tris-buffered saline, 0.1% Tween 20) containing 5% (w/v) BSA, and probed with the appropriate primary antibodies: anti-corin (1:1,000, rabbit, ab125254, Abcam), anti-PCSK6 (1:1,000, rabbit, ab151562, Abcam), anti-furin (1:1,000, rabbit, ab183495, Abcam), anti-ADAM10 (1:2,000, rabbit, ab124695, Abcam), anti-neprilysin (1:2,000, rabbit, ab126593, Abcam), anti-ANP (1:1,000, rabbit, ab209232, Abcam), anti-BNP (1:500, rabbit, ab19645, Abcam), and anti-GAPDH (1:500, mouse, sc-32233, Santa Cruz) diluted in blocking solution. After washing with TBS-T, the immunoreactive proteins were visualized with horseradish-conjugated goat anti-rabbit (1:25,000, 111-035-144, Jackson) and donkey anti-mouse (1:10,000, 715-035-151, Jackson) IgG secondary antibodies and chemiluminescent substrate.

### Gene Expression Analysis by Real-Time qPCR

Total RNA was isolated from snap-frozen tissue samples using TRIzol^®^ Reagent (Life Technologies), according to the instructions of the manufacturer, and quantified by spectrophotometry using NanoDrop2000. After oligo (dT)–primed reverse transcription of 1,000 ng total RNA, the resulting single-stranded cDNA was used for PCR. PCR conditions were as follows: an initial denaturation step at 95°C for 3 min, 30 cycles of denaturation at 95°C for 30 s, and hybridization at 60°C for 30 s followed by elongation at 72°C for 1 min. Finally, the PCR reaction was terminated by incubation at 72°C for 5 min. GAPDH was used as an internal standard. The following primers were used:

Corin: F(5′-GAAGACTGTAAGGACGGGAGTGA-3′),

 R(5′-GTCAAGGCAACCCCGATCT-3′);

PCSK6: F(5′-GCTCACGGCTACCTCAACTT-3′),

 R(5′-CTGTCTCTTGACCCTGCGTT-3′);

Furin: F(5′-AGGGGTAGGCTGACATCATCT-3′),

 R(5′-CCAGGGCACAGTGTTAGTTTG-3′);

ADAM10: F(5′-TGGTGTTGCCGACAGTGTTA-3′),

 R(5′-GGATTTCCATACTGACCTCCCA-3′);

Neprilysin: F(5′-ACACATGACCAAATAAACCATTGCT-3′),

 R(5′-GCTCACCCCAGAGTTTGTGT-3′);

NPPA: F(5′-CCTGGACTGGGGAAGTCAAC-3′),

 R(5′-ATCTATCGGAGGGGTCCCAG-3′);

NPPB: F(5′-TTTCCTTAATCTGTCGCCGCT-3′),

 R(5′-TGGATTGTTCTGGAGACTGGC-3′);

GAPDH: F(5′-GTGCCAGCCTCGTCTCATAG-3′),

 R(5′-GAGAAGGCAGCCCTGGTAAC-3′).

### Statistical Analysis

Data are presented as mean ± SEM. Comparison between two parametric groups was performed using the unpaired Student’s *t*-test after testing for equality of variances. A value of *p* < 0.05 was considered statistically significant.

## Results

As previously reported, rats with ACF exhibited two distinctly different patterns of UnaV. While some of the animals displayed progressive sodium retention, severe dyspnea, and edema, characteristics of decompensated CHF, the remaining rats with ACF, termed compensated subgroup, displayed increased UNaV comparable with those measured in the control group (Khoury et al., 2018). Bodyweight decreased in the compensated (from 343 ± 10 to 328 ± 6 gr) and a more pronounced manner in the decompensated CHF rats (from 339 ± 3 to 294 ± 4 gr). The placement of ACF caused an overt increase in the heart weight due to marked left and right ventricular hypertrophy/dilation (Supplementary Table S1). The heart/bodyweight ratio, an index of cardiac hypertrophy, was significantly elevated after 1 week of ACF surgery in the compensated and decompensated CHF groups relative to the sham rats (0.44 ± 0.01% and 0.50 ± 0.02% vs. 0.29 ± 0.004%, *P* < 0.01, respectively). The significant increase in HW/BW in the decompensated subgroup as compared with compensated animals, but not in the absolute HW, may partially stem from the body weight loss, which was more prominent in the decompensated group (−13.1 ± 1.2%) than the compensated group (−4.15 ± 2.6%), as compared with sham-operated rats, which gained 1.3 ± 3.5% in their body weights. In contrast to the cardiac tissue, kidney weight normalized to body weight were lower in rats with compensated CHF (0.3 ± 0.01%; *P* < 0.01) and to a larger extent in decompensated CHF (0.27 ± 0.01%; *P* < 0.001) as compared with the sham group (0.33 ± 0.01%) (Supplementary Table S1). Cardiac and renal remodeling was accompanied by lung edema, as previously reported (Supplementary Table S1). Furthermore, rats with CHF showed attenuated renal hemodynamic and impaired kidney function, as was evident by reduced RBF and GFR in correlation with the severity of the disease (Khoury et al., 2018). In addition, our former study demonstrated that rats with CHF displayed significant elevation in the circulating levels of ANP, which was comparable in compensated and decompensated CHF, compared with sham controls (Khoury et al., 2018). As expected, BNP levels in the circulation were significantly elevated in rats with compensated and decompensated CHF by 6- and 14-fold compared with sham-operated animals. All these findings indicate that the applied ACF model exhibits typical manifestations of CRS and composes a reliable platform to study this clinical setting.

### Cardiac Hemodynamics

**FIGURE 1:**
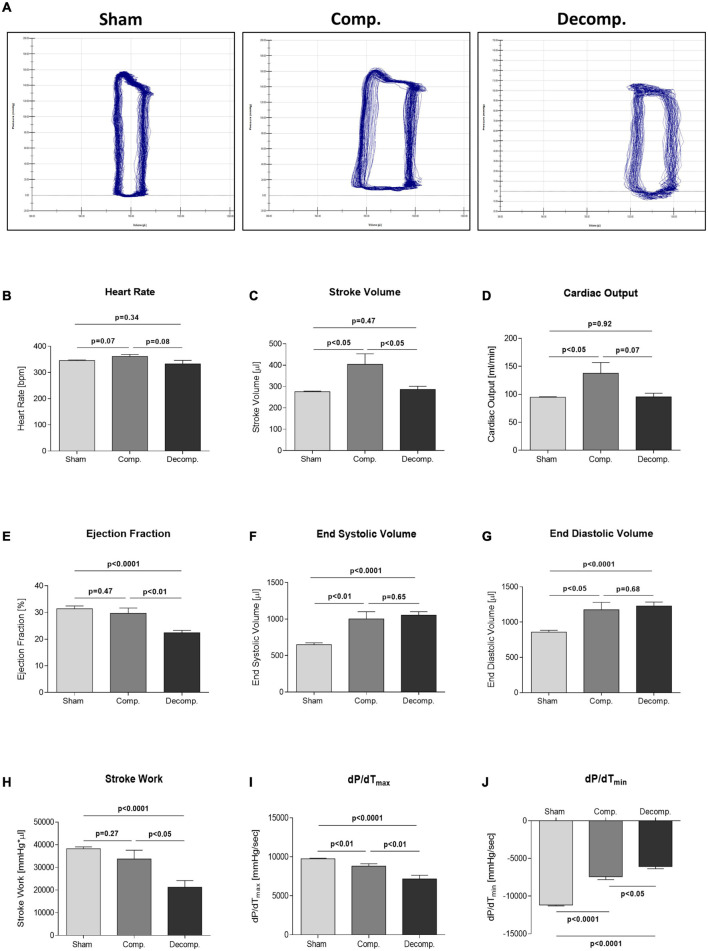
Pressure-volume loops in compensated and decompensated CHF and their sham controls. **(A)** Representative pressure-volume loops of rats with compensated CHF, decompensated CHF, and sham controls. **(B)** Heart rate, **(C)** stroke volume, **(D)** cardiac output, **(E)** ejection fraction, **(F)** end-systolic volume, **(G)** end-diastolic volume, **(H)** stroke work, **(I)** dP/dT_*max*_, **(J)** dP/dT_*min*_. Ejection fraction equals (end-diastolic volume – end-systolic volume)/end diastolic volume. Stroke work is calculated as the area under the pressure-volume curve (volume*pressure product). Maximum and minimum dP/dt signify the maximal systolic pressure generation as a parameter of left ventricular contractility and the minimal diastolic pressure loss as a parameter of left ventricular relaxation capacity, respectively. Values are means ± SEM.

Representative PV loops of compensated and decompensated subgroups and their sham controls are presented in Figure 1. Compensated and decompensated CHF rats exhibited significantly increased end-diastolic volumes as compared with sham operated controls (1,176 ± 104 μl; *P* < 0.05, 1,228 ± 57 μl; *P* < 0.0001 vs. 859 ± 25 μl, respectively) (Figure 1G). CHF rats also had increased end-systolic pressure, a parameter of LV wall stress (Figure 1F). As expected in the applied model, compensated but not decompensated CHF rats, displayed significantly increased stroke volume (404 ± 49 μl; *P* < 0.05) and cardiac output (138 ± 19 ml/min; *P* < 0.05) compared with controls (276 ± 3 μl and 95 ± 1.1 ml/min, respectively) (Figures 1C,D), but still having comparable heart rate (Figure 1B). Moreover, LV ejection fraction was unaltered in compensated CHF rats but significantly reduced in decompensated animals, as compared with the control group (29.7 ± 2%; *P* = NS, 22.4 ± 0.9%; *P* < 0.0001 vs. 31.4 ± 1.1%, respectively) (Figure 1E). Also, the systolic parameter of stroke work was significantly decreased in CHF rats in correlation with the severity of HF (Figure 1H). Finally, the maximal and minimal pressure gradients corresponding to systole and diastole, parameters of systolic and diastolic function, respectively, were reduced in rats with CHF (Figures 1I,J).

### Cardiac and Renal Fibrosis

**FIGURE 2:**
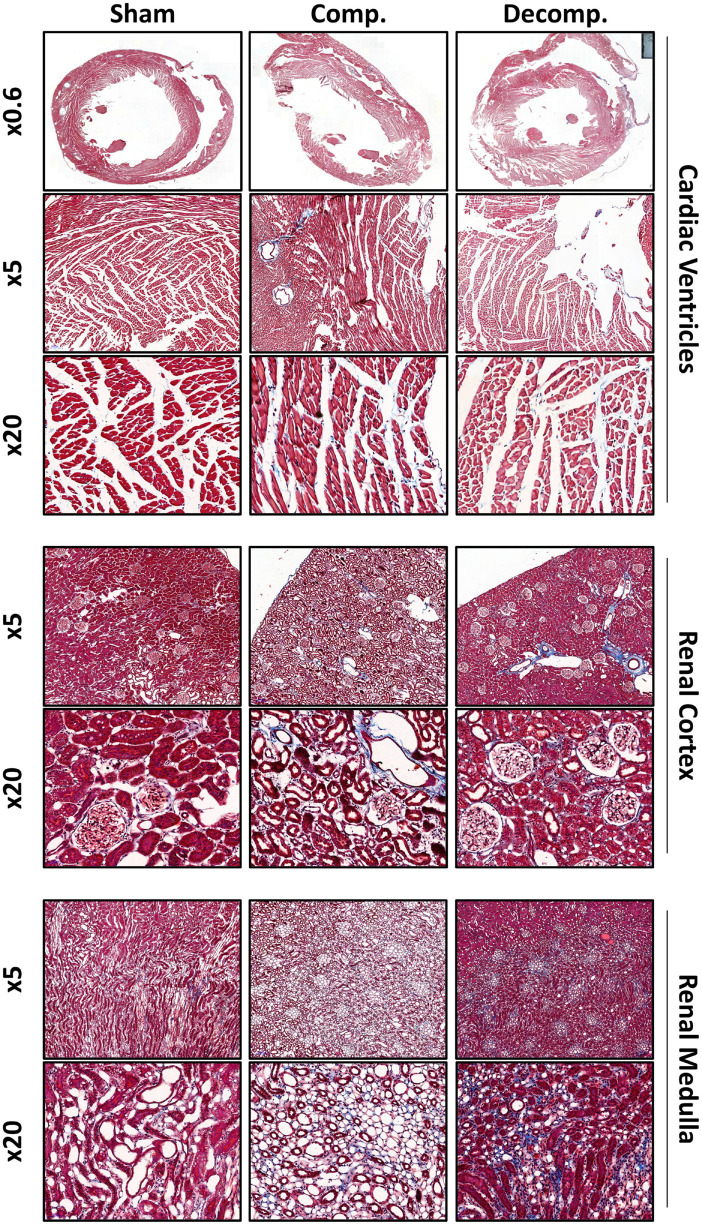
Tissue fibrosis in ACF rats. Representative images of Masson’s trichrome staining of the cardiac ventricular and renal tissues, as well as renal cortical and medullar layers, illustrating the impact of ACF placement on the development of tissue remodeling, compared with sham operated rats. Images are presented at x0.6, x5, and x20 magnification.

Figure 2 shows representative images of Masson’s trichrome staining depicting interstitial fibrosis in cardiac ventricular tissue and also the cortical and medullary layer of the kidney. There was little, blue-stained fibrotic tissue in sham-operated hearts, but fibrosis was apparent in both compensated and decompensated CHF hearts, especially in the endocardium layer and perivascular areas. Interstitial fibrosis is an additional consequence of cardiac remodeling in CHF rats, where cardiac fibrosis was more prominent in decompensated rats than that observed in compensated animals. Similarly, renal fibrosis localized mainly to the medullary and perivascular regions was aggravated by decompensated CHF, but to a lesser extent in the compensated subgroup. These results suggest that besides cardiac hypertrophy and kidney dysfunction, CHF is associated with cardiac and renal fibrosis, yet it was very mild probably due to the short follow-up period (1 week) after the induction of the disease. In addition, trichrome staining can also stain the tissue with blue color not only in the presence of fibrosis but also secondary to edema, which is a hallmark feature of HF. Finally, the renal medulla is a collagen-rich tissue, and thus, the obtained trichrome staining may represent basal rather than evolving fibrosis.

### Cardiac and Renal Expression of Corin/PCSK6 in Rats With Aorto-Caval Fistula

#### Heart

**FIGURE 3:**
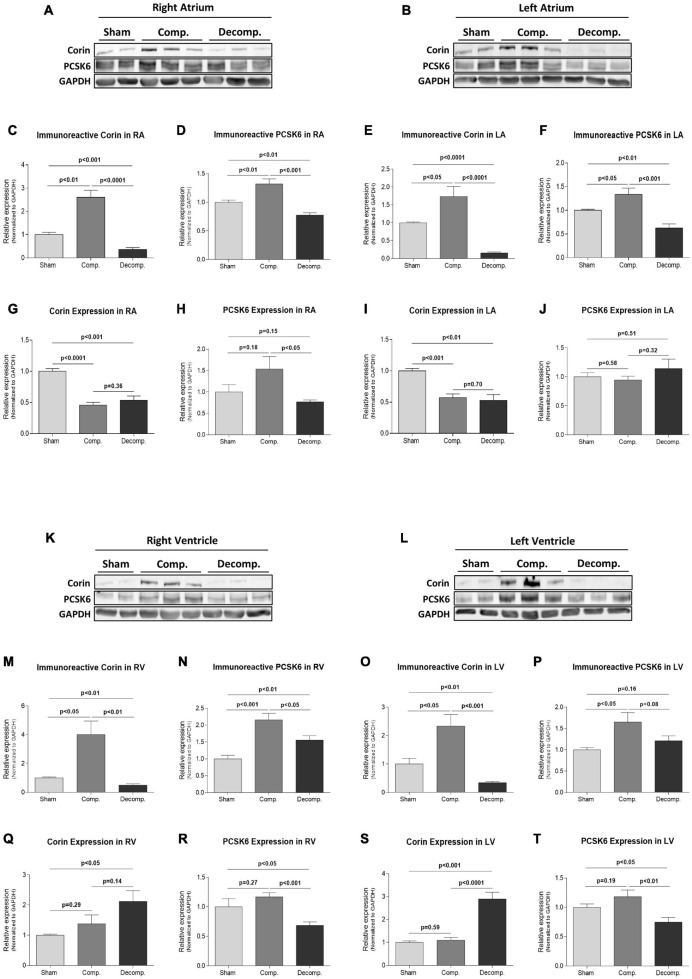
Levels and expression of corin and PCSK6 in the cardiac tissue of compensated CHF, decompensated CHF, and sham controls. Representative western blot analysis of corin and PCSK6 in RA, LA, RV, and LV tissue lysates are shown in **(A,B,K,L)**, respectively. **(C–F,M–P)** Represent western blot analysis relative quantification of corin and PCSK6 in the different heart chambers, where GAPDH was used as a loading control. Quantification of qPCR analysis for corin and PCSK6 mRNA normalized to GAPDH are depicted in **(G–J,Q–T)**. Values are means ± SEM.

As shown in Figure 3, rats with decompensated HF exhibit a severe reduction in the immunoreactive relative levels of corin in all cardiac chambers, as compared with both compensated HF and sham-operated groups (Figures 3C,E,M,O). In contrast, the compensated subgroup displayed an enhanced abundance of corin in both atrial and ventricular tissues. Specifically, the relative expression of corin immunoreactivity in RA, LA, RV, and LV of decompensated rats was 0.36 ± 0.08 (*P* < 0.0001), 0.16 ± 0.02 (*P* < 0.0001), 0.49 ± 0.1 (*P* < 0.01), and 0.34 ± 0.04 (*P* < 0.001), as compared with 2.61 ± 0.3, 1.74 ± 0.28, 4.01 ± 0.94, 2.33 ± 0.41, in compensated animals, respectively. While corin mRNA expression in the atria of decompensated rats followed a similar pattern, the compensated subgroup also showed downregulation of corin mRNA (Figures 3G,I). Interestingly, corin transcript levels in the right and left ventricular tissues were significantly elevated in decompensated HF (2.12 ± 0.36 and 2.89 ± 0.29, respectively) compared with sham controls (1.0 ± 0.04; *P* < 0.05 and 1.0 ± 0.06; *P* < 0.001, respectively) and compensated subgroup (1.38 ± 0.29; *P* = NS, and 1.1 ± 0.13; *P* < 0.0001, respectively) (Figures 3Q,S).

Concerning PCSK6 abundance, both right (0.78 ± 0.04) and left (0.63 ± 0.09) atrium of decompensated HF animals displayed a significant decrease in immunoreactive levels of the enzyme, as compared with compensated HF animals (1.32 ± 0.09; *P* < 0.001 and 1.34 ± 0.13; *P* < 0.001, respectively) and sham-operated rats (1.0 ± 0.04; *P* < 0.01 and 1.0 ± 0.02; *P* < 0.01, respectively) (Figures 3D,F). Similarly, PCSK6 abundance was upregulated in right (2.2 ± 0.2;*P* < 0.001) and left (1.65 ± 0.22; *P* < 0.05) ventricles of compensated HF rats, but to a lesser extent in decompensated subgroup (1.55 ± 0.13; *P* < 0.01 and 1.21 ± 0.12; *P* = NS, respectively), when compared with sham controls (1.0 ± 0.1 and 1.0 ± 0.05, respectively) (Figures 3N,P). While PCSK6 mRNA expression in RA (0.77 ± 0.05; *P* = NS) and RV (0.69 ± 0.06; *P* < 0.05) of the decompensated rats was attenuated, it increased in the compensated subgroup (1.54 ± 0.29; *P* = NS and 1.17 ± 0.07; *P* = NS, respectively) as compared with sham controls (1.0 ± 0.17 and 1.0 ± 0.14, respectively) (Figures 3H,R).

**FIGURE 4:**
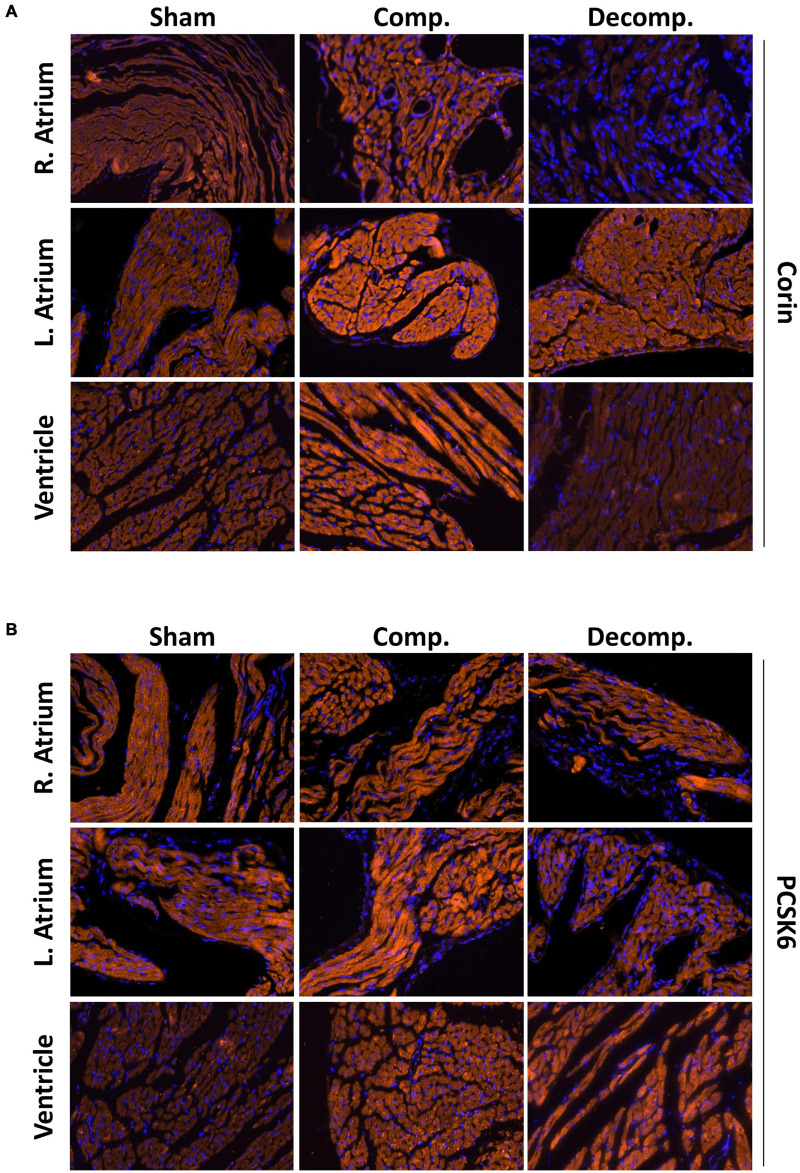
Corin/PCSK6 staining in the heart chambers of ACF and sham-operated rats. Representative images of immunofluorescence staining for corin **(A)** and PCSK6 **(B)** in the cardiac atrial and ventricular tissues of rats with compensated CHF, decompensated CHF, and sham-operated animals. Tissues were counterstained with DAPI. All images were obtained at x40 magnification.

Finally, the distinct patterns of myocardial PCSK6/corin behavior in rats with compensated and decompensated CHF were also confirmed by immunofluorescence analysis. Immunofluorescence staining revealed intense immunostaining of corin in the cardiac and renal tissues (Figure 4A). In the myocardium, corin immunofluorescence was detected mainly in the myocytes, both at the cellular membrane and cytosol. In agreement with WB analysis, immunofluorescence of cardiac corin was enhanced in compensated CHF group, but not in decompensated CHF animals. Cardiac PCSK6 immunofluorescence followed a similar pattern as corin, namely, more abundant in compensated subgroup but not decompensated animals (Figure 4B). It should be emphasized that PCSK6 was localized mainly to the myocytes.

Collectively, Western blot, RT-qPCR, and immunofluorescent analysis revealed upregulation of corin and PCSK6 in the myocardium of rats with compensated CHF week, but downregulation of these two enzymes in the decompensated subgroup.

#### Kidney

**FIGURE 5:**
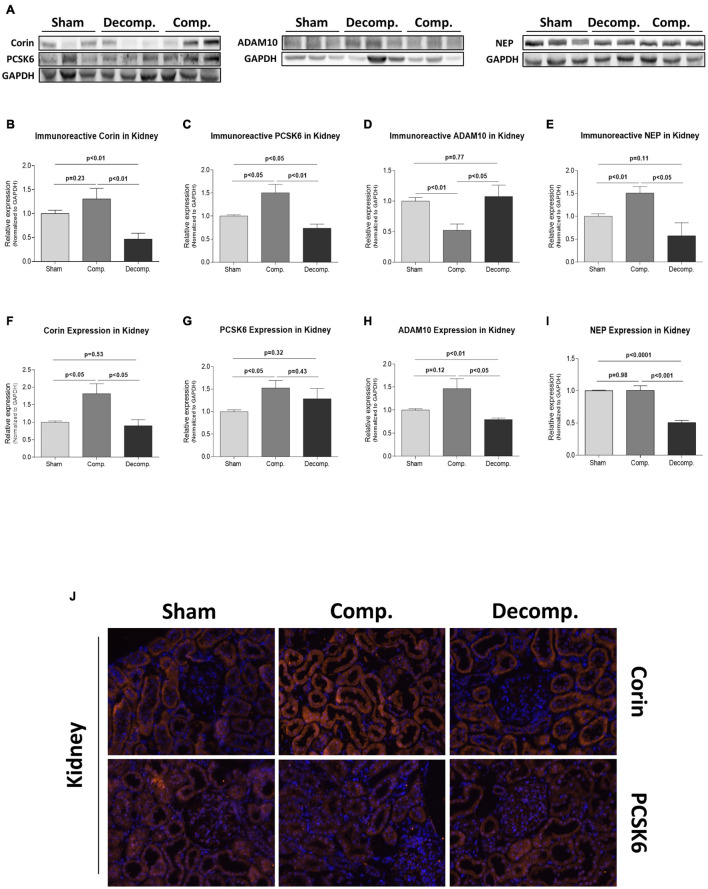
Natriuretic peptide machinery system in the renal tissue of ACF and sham-operated rats. Representative western blot analysis **(A)** with antibodies against corin, PCSK6, ADAM10, and NEP and their quantification relative to GAPDH **(B–E)** in the kidney of ACF rats and their sham operated rats. **(F–I)** represents quantification of qPCR analysis of the mRNA of these enzymes relative to GAPDH. **(J)** Representative images of immunostaining of corin and PCSK6 in the kidney of the various animal groups, counterstained with DAPI. All images were obtained at x40 magnification. Values are presented as means ± SEM.

Figure 5 depicts the changes in the abundance and expression of both corin (Figures 5B,F) and PCSK6 (Figures 5C,G) in the renal tissue of compensated and decompensated CHF rats as compared with sham controls. Akin to the myocardium, decompensated rats exhibited a decline in renal corin immunoreactivity and mRNA transcript (0.47 ± 0.13; *P* < 0.01 and 0.9 ± 0.17; *P* = NS, respectively) as compared with their controls (1.0 ± 0.07 and 1.0 ± 0.04, respectively), whereas compensated CHF displayed upregulation of this enzyme as demonstrated by both the immunoreactive and mRNA levels (1.31 ± 0.22; *P* = NS and 1.94 ± 0.32; *P* < 0.05, respectively). In line with these changes, PCSK6 abundance was significantly increased in the compensated subgroup (1.5 ± 0.18; *P* < 0.05), but reduced in decompensated animals (0.74 ± 0.09; *P* < 0.05), as compared with sham-operated rats (1.0 ± 0.04) (Figure 5C), whereas the expression of PCSK6 was increased in both compensated and decompensated CHF rats (1.52 ± 0.17; *P* < 0.05 and 1.28 ± 0.23; *P* = NS, respectively), when compared with the control group (1.0 ± 0.04) (Figure 5G).

Immunofluorescent analysis of the kidney unraveled the abundance of corin immunoreactivity in the proximal tubule, mTAL, and to a lesser extent in the collecting duct. In the proximal tubule, corin was localized to the apical membrane underneath the brush border and a lesser extent in the cytosol. Interestingly, immunofluorescent staining revealed a remarkable increase of corin in the renal tissue of compensated rats, but a reduction of this enzyme in decompensated animals (Figure 5J).

### Cardiac and Renal Expression of ADAM10 in Rats With Aorto-Caval Fistula

**FIGURE 6:**
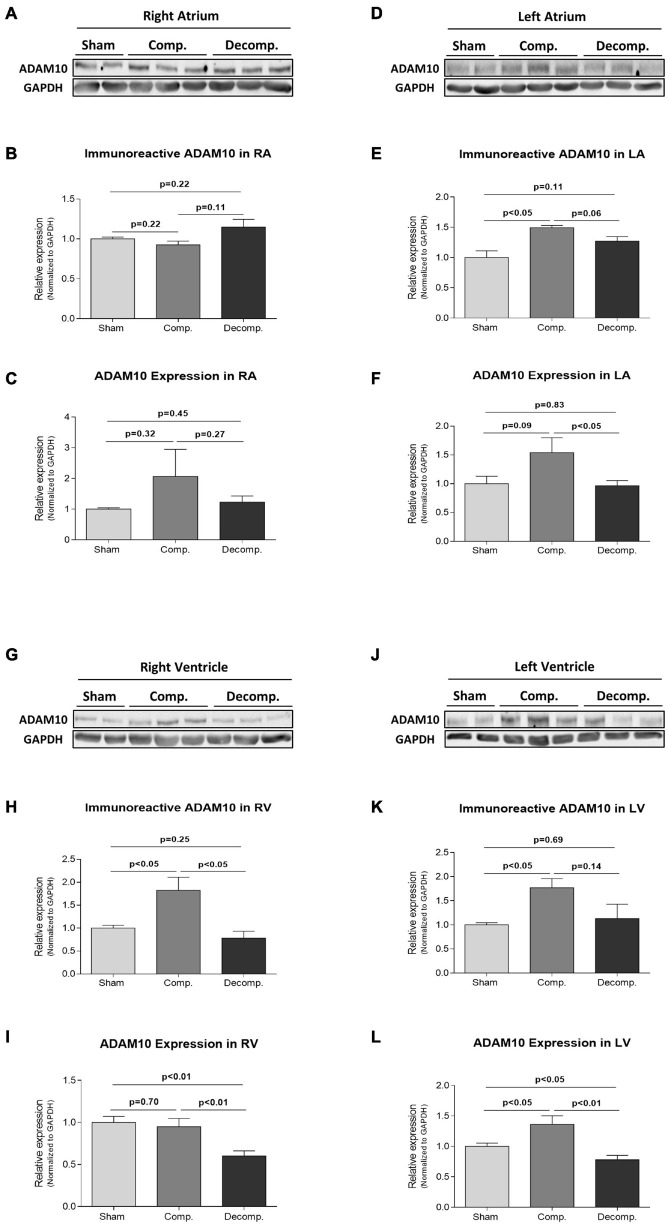
Abundance and expression of ADAM10 in heart chambers of ACF and sham-operated rats. Representative western blot analysis of ADAM10 **(A,D,G,J)** and its quantification relative to GAPDH **(B,E,H,K)** in RA, LA, RV, and LV, respectively, of ACF and sham-operated rats. **(C,F,I,L)** Represent quantification of qPCR analysis of ADAM10 mRNA relative to GAPDH in the heart chambers of the different animal groups. Values are presented as means ± SEM.

Since corin is regulated by shedding and releasing to the blood, either by autocleavage or by ADAM10-mediated cleavage (Jiang et al., 2011), we also studied the behavior of this enzyme in the heart and kidneys of CHF rats as compared with sham-operated animals. Representative western blot analysis of cardiac ADAM10 in RA, LA, RV, and LV chambers are presented in Figures 6A,D,G,J, respectively. In contrast to RA (0.93 ± 0.05; *P* = NS), ADAM10 immunoreactivity was increased in LA (1.49 ± 0.04; *P* < 0.05), RV (1.82 ± 0.29; *P* < 0.05) and LV (1.77 ± 0.19; *P* < 0.05) (Figures 6B,E,H,K, respectively) of compensated rats compared to their controls (1.0 ± 0.02, 1.0 ± 0.11,1.0 ± 0.06, and 1.0 ± 0.04, respectively). Noteworthy, the abundance of ADAM10 did not change in the various cardiac chambers of decompensated rats. The expression of ADAM10 followed a similar pattern as the immunoreactive levels, i.e., upregulation in RA, LA, and LV (Figures 6C,F,L, respectively), but not RV (Figure 6I). Specifically, ADAM10 mRNA was downregulated in the RV and LV of the decompensated rats, but it did not change in the atrial tissues. At the renal level, ADAM10 immunoreactivity was significantly reduced in compensated rats (0.52 ± 0.1; *P* < 0.01) but did not change in the decompensated subgroup (1.08 ± 0.19; *P* = NS), compared with control animals (1.0 ± 0.06) (Figure 5D). ADAM10 expression was significantly increased in compensated CHF rats (1.47 ± 0.21; *P* = NS) but decreased in decompensated animals (0.79 ± 0.03; *P* < 0.01), as compared with sham-operated rats (1.0 ± 0.03) (Figure 5H).

### Renal Expression of Neprilysin in Rats With Aorto-Caval Fistula

Since NEP is a key enzyme in the degradation of NPs (Kenny et al., 1993; Watanabe et al., 1997), its abundance and expression in the renal tissue were also determined. Figures 5E,I depict the expression and abundance of this enzyme in the renal tissue of the studied experimental groups, respectively. While NEP immunoreactivity was significantly increased in the kidney of compensated CHF animals (1.51 ± 0.51; *P* < 0.01), it slightly decreased in the decompensated animals (0.57 ± 0.28; *P* = NS) compared with controls (1.0 ± 0.05). NEP expression was significantly decreased in decompensated animals (0.51 ± 0.03; *P* < 0.0001) but did not change in the compensated subgroup (1.0 ± 0.07; *P* = NS), as compared with shams (1.0 ± 0.01).

### Cardiac and Renal Expression of Atrial Natriuretic Peptide and Brain Natriuretic Peptide in Rats With Aorto-Caval Fistula

**FIGURE 7:**
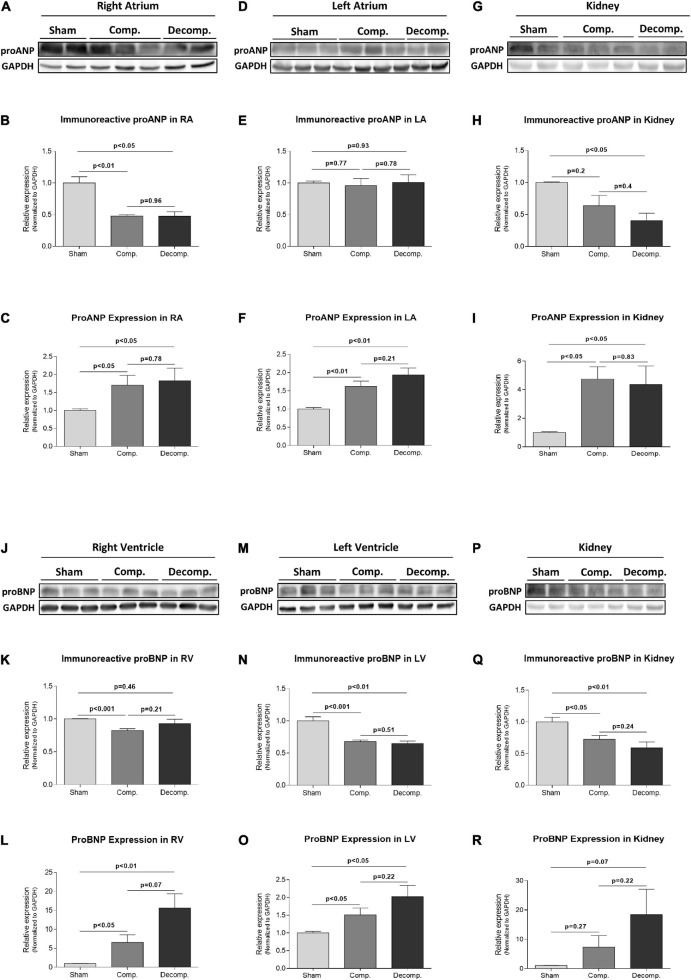
Abundance and expression of atrial and B-type natriuretic peptides in the cardiac and renal tissues of ACF and sham-operated rats. Representative western blot analysis of proANP **(A,D,G)** and proBNP **(J,M,P)** and their quantification relative to GAPDH **(B,E,H,K,N,Q)** in heart chambers and kidneys of ACF and sham-operated rats. **(C,F,I,L,O,R)** Represent quantification of qPCR analysis of proANP and proBNP mRNA relative to GAPDH in the same tissues. Values are presented as means ± SEM.

After unraveling the presence of Corin/PCSK6 transcripts, we examined the expression and abundance of proANP and proBNP hormones in the atrial and ventricular myocardial chambers, respectively, and also in the renal tissue of the various experimental groups. Figures 7A,D,G depict representative western blot analysis of proANP in RA, LA, and renal tissues of compensated and decompensated ACF-operated rats, as compared to control animals, respectively. While the immunoreactive levels of proANP decreased in RA (Figure 7B) in both compensated and decompensated CHF rats (0.48 ± 0.02; *P* < 0.01, 0.48 ± 0.07; *P* < 0.01 vs. 1.0 ± 0.01, respectively), but not in LA of rats with CHF (Figure 7E), the expression of proANP increased in both RA and LA as a function of CHF severity (Figures 7C,F). A similar pattern was obtained also in renal proANP abundance, where it declined in CHF subgroups (Figure 7H). The downregulation of proANP abundance in the kidney was associated with the upregulation of its renal expression in both subgroups of CHF rats (Figure 7I). Representative western blot analysis of proBNP in RV, LV, and renal tissues are shown in Figures 7J,M,P, respectively. Applying western blot analysis for proBNP in the cardiac ventricles (the major site of BNP production) revealed attenuated levels of proBNP immunoreactivity in the LV of both compensated and decompensated CHF rats (0.68 ± 0.02; *P* < 0.001, 0.65 ± 0.04; *P* < 0.01 vs. 1.0 ± 0.06, respectively), but not RV (Figures 7K,N). In contrast, the expression of proBNP in both RV and LV was enhanced in rats with ACF in correlation with the severity of CHF (Figures 7L,O). In line with the alterations in renal proANP, immunoreactive proBNP in the kidney displayed a similar pattern; namely, it declined in CHF subgroups (Figure 7Q), but its expression was augmented in these animals (Figure 7R).

## Discussion

The current study sheds light on the status of corin and PCSK6 in the cardiac and renal tissues of rats with CHF of various severities. As we have demonstrated in several studies, rats with ACF display cardiac hypertrophy, pulmonary congestion, and impaired kidney function, and renal hypoperfusion, which are typical manifestations of CHF (Abassi et al., 1990, 2001, 2011; Khoury et al., 2018). In this context, placement of ACF caused an overt increase in heart/body weight (BW), an index of cardiac hypertrophy, which was more evident in decompensated CHF after 1 week from surgery. In addition, rats with ACF exhibited reduced cardiac performance as a function of the severity of the disease. Moreover, absolute and normalized lung weight to BW of CHF rats was also higher than those of controls. In contrast, kidney weight normalized to BW was lower in both subgroups of CHF rats, namely compensated and decompensated, as compared with sham-operated rats. These features support the reliability of ACF as a CRS experimental model.

Along with these renal and cardiac hemodynamic alterations, the present study clearly shows that corin immunoreactive levels were significantly increased in all cardiac chambers of compensated but decreased in decompensated CHF. In line with these findings, the immunofluorescent analysis revealed upregulation and downregulation of myocardial corin immunoreactive protein in compensated and decompensated CHF, respectively. The pattern of corin staining strongly suggests cytosolic and myocyte membranal localization within cells that also contain proANP and proBNP (Hooper et al., 2000; Bialik et al., 2001; Gladysheva et al., 2008). This pattern supports the concept that corin is transported from the intracellular compartment to the cell membrane, where it converts proANP and proBNP to ANP and BNP upon secretion (Wu et al., 2009). Concomitant with the alterations in cardiac corin abundance, corin mRNA was detected in the myocardium of the various experimental groups, where its expression was downregulated in the atrial tissues of both compensated and decompensated rats. In contrast, the expression of corin was upregulated in the ventricular tissues of decompensated subgroup and to a lesser extent in the compensated one.

Concerning PCSK6, our results revealed that CHF is associated with changes in this key enzyme, corresponding to corin behavior. Specifically, PCSK6 increased in all heart chambers in compensated CHF, yet significantly decreased in the atrial tissues and RV but to a lesser extent in the LV of the decompensated subgroup. Finally, the present study unequivocally demonstrates the existence of corin and PCSK6 in the renal tissues as was evident by WB, PCR, and immunohistochemical analysis, especially in the proximal tubule and the collecting duct. Interestingly, compensated CHF was associated with a slight increase of these two enzymes, whereas decompensated CHF exhibited a decline in both the renal corin and PCSK6, suggesting a role of locally produced NPs in the regulation of salt/water excretion on one hand and that perturbations in this system may play a role in the pathogenesis of Na+ retention and edema formation during the decompensated stage.

The present study extends previously published research that examined the changes in myocardial corin and its relevance to the pathogenesis of HF. In this regard, patients with decompensated HF displayed lower levels of circulating corin along with decreased cleavage of proANP (Ibebuogu et al., 2011). In agreement with these findings, Langenickel et al. (2004) showed that corin declined in the atrium 4 weeks after CHF induction by a similar surgical maneuver applied in the present study, namely ACF (Langenickel et al., 2004). In contrast, overexpression of corin mRNA was obtained in the non-infarcted LV myocardium after 8 weeks from the induction of hypertrophied ventricle induced by left anterior descending (LAD) artery ligation in rats (Tran et al., 2004). Unfortunately, the clinical study focused on circulating corin (Langenickel et al., 2004), whereas in the LAD model the authors measured mRNA corin, but not immunoreactive levels (Tran et al., 2004). Thus, our approach of measuring both the expression and abundance of corin simultaneously in the various heart chambers and the kidney provides a more comprehensive picture of the status of this enzyme in rats with CHF of various severities.

Our findings that corin immunoreactivity and expression in the various heart compartments increased in the compensated and declined in decompensated subgroup could be of clinical relevance. Considering that corin plays a beneficial role against the development of either dilated cardiomyopathy (DCM) or reduced ejection fraction, HF, and regulation of salt homeostasis (Tripathi et al., 2019), upregulation of cardiac corin in compensated CHF rats may play a role in maintaining normal Na^+^ balance, whereas the decline in this key enzyme in decompensated subgroup likely aggravates salt retention, edema formation, and cardiac hypertrophy. Support for this concept is derived from the findings that reduced plasma corin levels have been reported in patients with HF, and the magnitude of reduction in plasma corin levels correlates with HF severity (Dong et al., 2010; Ibebuogu et al., 2011). In addition, cardiac-specific expression of catalytically active corin delays the onset of symptomatic CHF associated with lung edema and extends life in the experimental mouse model of DCM (Gladysheva et al., 2013; Ngo et al., 2013). Moreover, upregulation of cardiac corin mRNA was observed in the cardiac tissue of heart transplant recipients with preserved cardiac function in comparison with DCM and reduced ejection fraction HF (Verstreken et al., 2019). Collectively, these findings indicate that enhanced levels of locally cardiac corin levels might delay the progression of cardiac dysfunction and remodeling.

Our study also examined the changes in PCSK6, the primary activating enzyme of corin, in the various experimental groups. The current results demonstrate that the changes in myocardial corin correspond with PCSK6 alterations (Chen et al., 2015). These changes in the PCSK6/corin/ANP-BNP pathway have adverse physiological consequences which affect the production of ANP/BNP from proANP/proBNP, respectively, and eventually resulting in the disruption in sodium balance and cardiac remodeling (Ibebuogu et al., 2011). Indeed, corin deficiency in mice was associated with reduced sodium excretion and salt sensitive hypertension and cardiac hypertrophy (Chan et al., 2005; Wang et al., 2012). Likewise, PCSK6-deficient mice display salt-sensitive hypertension (Chen et al., 2015). Our findings that PCSK6/corin decreased in decompensated rats but increased in compensated ones may contribute to the enhanced cardiac hypertrophy and decline in Na^+^ excretion in the former but not the latter. The sustained elevation of ANP/BNP plasma levels despite the decline in both PCSK6/corin in our rats could be attributed to assumedly sufficient levels of the remnant two enzyme or alternatively to the detached circulatory PCSK6/corin which retains proteolytic activity and is capable of maintaining enhanced production of NPs.

One of the main novel aspects of the present study is the exploration of corin/PCSK6 status in the renal tissue of compensated and decompensated CHF animals. Expression of corin has been documented in both animal (Polzin et al., 2010) and human kidneys (Langenickel et al., 2004; Tran et al., 2004) in the same localizations as those observed in our study. The status of corin in the renal tissue of rats with CHF was not studied yet. Our findings revealed upregulation and downregulation of corin in compensated and decompensated CHF, respectively. These results suggest a role of corin in kidney function, both in health and in renal dysfunction, under edematous disease states such as CHF, as this machinery may be responsible for the local conversion of proANP to ANP, which in its turn act in an autocrine manner to regulate sodium/water reabsorption. Besides CHF, Polzin et al. (2010) have demonstrated a downregulation in renal corin along with severe proteinuria and salt/water retention in an experimental model of nephrotic syndrome. Also in this study, the immunoreactive corin was localized to the proximal tubule, mTAL, and collecting duct. The latter is a well-known site for ANP action, and assumedly, ANP production. Specifically, proANP, natriuretic peptide receptor-A (NPR-A) mRNA, and their immunoreactive peptides were detected in this segment of the nephron (Zeidel, 1990; Polzin et al., 2010; Ichiki et al., 2011; Dong et al., 2016). Our discovery that proximal tubule and mTAL also expresses corin is in line with similar findings in specimens of human kidney (Dong et al., 2016) and strongly suggests that this nephron segment may be a major site of ANP production. Concerning the proximal tubule, a previous study demonstrated that proANP and NPR-A mRNA are also expressed in this segment of the tubule even more than the collecting duct (Dong et al., 2016). Interestingly, renal immunoreactive levels of both proANP and proBNP were declined in rats with compensated CHF and to a larger extent in the decompensated subgroup. The significant upregulation in the expression of these NPs in the renal tissue may represent a compensatory response to the decline in the immunoreactive peptides.

Additional enzyme that may affect the local and circulatory levels of corin is ADAM10. ADAM10 is responsible for corin shedding, thus affecting the abundance of corin on the cellular membrane, as well as its plasma levels. In this context, soluble corin was detected in the blood of normal subjects, and its levels were significantly reduced in patients with severe HF as compared with healthy subjects and even patients with mild cardiac dysfunction (Dong et al., 2010; Ibebuogu et al., 2011). These findings suggest that corin shedding from the cells by ADAM10 may be an important mechanism for regulating corin function and that altered corin shedding and/or cleavage may play a role in the pathogenesis of HF (Jiang et al., 2011). In this context, the current study demonstrated that rats with compensated, but not decompensated CHF, were characterized by upregulation of ADAM10 in most of the cardiac chambers. In contrast, renal ADAM10 was downregulated in the compensated subgroup. Considering that ADAM10 causes shedding of an active corin fragment (Jiang et al., 2011), changes in cardiac and renal expression of ADAM10 may play a role in the regulation of corin abundance and subsequently cardiac remodeling/function and sodium balance. Moreover, corin shedding may constitute a physiological approach aimed at preventing excessive, potentially hazardous, proteolytic activities in the heart.

Since plasma levels of ANP/BNP are not only affected only by corin but also by NEP, an enzyme responsible for ANP/BNP degradation, renal abundance/expression of NEP was determined in the various experimental groups. While compensated CHF rats exhibited enhancement in NEP in the renal tissue, decompensated subgroup displayed a decline in this enzyme. The physiological significance of this phenomenon remains to be determined; however, it may represent a counterbalance response to the upregulation of corin in the compensated animals to avoid the exaggerated production of NPs.

Since furin is an additional key enzyme responsible for the activation of BNP, we previously examined the status of furin in the cardiac and renal tissues of compensated and decompensated animals and their controls (Khoury et al., 2021). Briefly, furin immunoreactivity in the LV was significantly enhanced in compensated and to a lesser extent in decompensated CHF subgroup, as compared with sham controls. A similar trend was detected in the RV of compensated, but not of decompensated CHF subgroup (Khoury et al., 2021). In line with these findings, the abundance and expression of furin were remarkably enhanced in the RA and LA of rats with CHF (Supplementary Figures S1A–F). Neither the abundance nor the expression of furin in the kidney has changed following the induction of CHF as was published recently (Khoury et al., 2021). Finally, similar to its behavior in cardiac tissue, both immunoreactive and expression of furin in the pulmonary tissue were significantly enhanced in correlation with CHF severity (Khoury et al., 2021).

In summary, we demonstrated similar cardiac and renal patterns of corin changes at the mRNA and protein levels as was evident by qPCR, WB, and immunohistochemistry analysis. Specifically, we found upregulation of corin and PCSK6 in the various heart chambers of compensated CHF but a decline in these two enzymes in the decompensated subgroup. Similarly, the application of the same molecular analysis to the kidney revealed comparable corin behavior as in cardiac tissue. Of note, in the kidney, the corin was abundant in the proximal tubule, mTAL, and collecting duct, suggesting that locally produced ANP may act as an autocrine mediator of salt/water homeostasis not only by acting on the CD but probably *via* proximal tubule and mTAL segments. We hypothesize that the obtained initial upregulation of cardiac and renal PCSK6/corin may represent a compensatory response to maintain Na^+^ balance, which subsequently fails as the disease progresses.

Yet, our study has few limitations: (1) It does not directly address mechanisms involved in the changes in the transcription and expression of corin and PCSK6 and the molecules linked to these enzyme regulations caused by CHF. Further studies are needed to assess their status in humans with CHF, and our relevant discussion is to a large extent speculative. Yet these assumptions and suggestions should be further evaluated, since they may bear clinical relevance with plausible interventional implications in this evolving disease; (2) additional limitation of the current study is the short follow up period (1 week), which represents acute CHF, rather than a chronic condition that usually develops in humans in many years; (3) we applied an acute CHF study carried out in an experimental model induced by a volume overload method, different from what is normally seen in humans (myocardial infarction or hypertension); this makes it difficult to translate the results to patients. (4) Normalized cardiac hypertrophy (HW/BW) was more evident in the decompensated subgroup; however, body weight loss in this subset of animals may have contributed to the calculated cardiac hypertrophy; (5) trichrome staining can stain tissues with blue color not only secondary to fibrosis, but also as a result of edema.

## Data Availability Statement

The original contributions presented in the study are included in the article/Supplementary Material, further inquiries can be directed to the corresponding author/s.

## Ethics Statement

The animal study was reviewed and approved by Experiments were performed according to the Guide for the Care and Use of Laboratory Animals (NIH Publication No. 85-23, revised 1996) as approved by the local institutional committee for supervision of animal experiments in the Technion.

## Author Contributions

EK: conceptualization, data collection and analysis, methodology, writing–original draft, and writing–review and editing. AF: data collection and analysis, methodology (immunostaining and RT-PCR). SK: methodology and software. YK: methodology (immunostaining) and software. DA: conceptualization, investigation (lead), writing–review and editing. ZA: conceptualization (lead), data collection, formal analysis (lead), funding acquisition (lead), investigation (lead), methodology (equal), project administration (lead), resources (lead), software, supervision (lead), validation (lead), writing–original draft (lead), and writing–review and editing (lead). All authors: contributed to the article and approved the submitted version.

## Conflict of Interest

The authors declare that the research was conducted in the absence of any commercial or financial relationships that could be construed as a potential conflict of interest.

## Publisher’s Note

All claims expressed in this article are solely those of the authors and do not necessarily represent those of their affiliated organizations, or those of the publisher, the editors and the reviewers. Any product that may be evaluated in this article, or claim that may be made by its manufacturer, is not guaranteed or endorsed by the publisher.

## Supplementary Material

Supplementary Figure S1 Abundance and expression of furin in the atria of ACF and sham-operated rats. Representative western blot analysis of furin **(A,D)** and its quantification relative to GAPDH **(B,E)** in RA and LA of ACF and sham-operated rats. **(C,F)** Represent quantification of qPCR analysis of furin mRNA relative to GAPDH in the same tissues. Values are presented as means ± SEM.

Supplementary Table S1 Impact of aortocaval placement on heart, lung, and kidney weights as compared with sham operated controls. Cardiac, lung, and kidney weights expressed either as absolute values or relative to bodyweight of rats with compensated and decompensated CHF and their sham controls. Values are means ± SEM. ^∗^*P* < 0.05; ^∗∗^*P* < 0.01; ^∗∗∗^*P* < 0.001; ^****^*P* < 0.0001 vs. sham-operated rats. ^†^*P* < 0.05; ^††^*P* < 0.01; ^††††^*P* < 0.0001 vs. compensated CHF group.
