# Supplementary figures and images for: Distribution of Cardiac and Renal Corin and Proprotein Convertase Subtilisin/Kexin-6 in the Experimental Model of Cardio-Renal Syndrome of Various Severities
Source: Front Physiol. 2021 Oct 18;12:673497. doi: 10.3389/fphys.2021.673497 (PMC8558519; doi:10.3389/fphys.2021.673497)

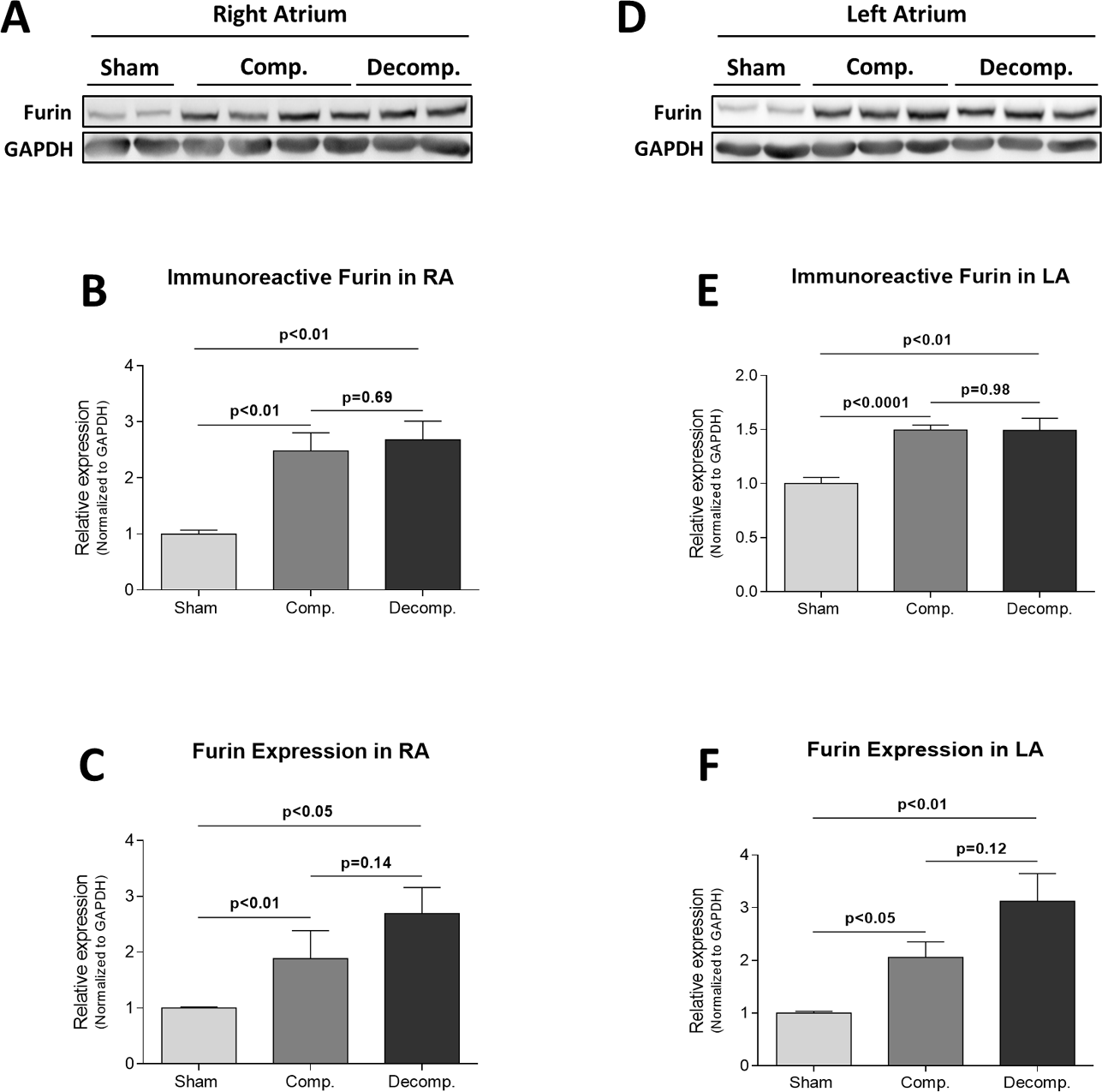

Supplement: Supplementary Figure S1 — Abundance and expression of furin in the atria of ACF and sham-operated rats. Representative western blot analysis of furin (A,D) and its quantification relative to GAPDH (B,E) in RA and LA of ACF and sham-operated rats. (C,F) Represent quantification of qPCR analysis of furin mRNA relative to GAPDH in the same tissues. Values are presented as means ± SEM. [file Image_1.TIF]
