# Supplementary material for: Distribution of Cardiac and Renal Corin and Proprotein Convertase Subtilisin/Kexin-6 in the Experimental Model of Cardio-Renal Syndrome of Various Severities
Source: Front Physiol. 2021 Oct 18;12:673497. doi: 10.3389/fphys.2021.673497 (PMC8558519; doi:10.3389/fphys.2021.673497)
Supplement: Supplementary Table S1 — Impact of aortocaval placement on heart, lung, and kidney weights as compared with sham operated controls. Cardiac, lung, and kidney weights expressed either as absolute values or relative to bodyweight of rats with compensated and decompensated CHF and their sham controls. Values are means ± SEM. ∗P < 0.05; ∗∗P < 0.01; ∗∗∗P < 0.001; ****P < 0.0001 vs. sham-operated rats. †P < 0.05; ††P < 0.01; ††††P < 0.0001 vs. compensated CHF group. [file Table_1.docx]

|  | **Sham** | **Compensated** | **Decompensated** |
| --- | --- | --- | --- |
| **Heart weight (g)** | 1.17 ± 0.02 | 1.48 ± 0.07 * | 1.48 ± 0.09 * |
| **Heart/Body weight ratio (%)** | 0.29 ± 0.004 | 0.44 ± 0.01 ** | 0.5 ± 0.02 ** |
| **Lung weight (g)** | 2.16 ± 0.1 | 2.35 ± 0.15 | 3.05 ± 0.13 ** † |
| **Lung/Body weight ratio (%)** | 0.65 ± 0.02 | 0.72 ± 0.03 | 1.04 ± 0.04 *** †† |
| **Kidney weight (g)** | 1.14 ± 0.02 | 1.02 ± 0.02 ** | 0.83 ± 0.03 **** †††† |
| **Kidney/Body weight ratio (%)** | 0.33 ± 0.01 | 0.3 ± 0.01 ** | 0.27 ± 0.01 *** † |

**Table S1: Impact of aortocaval placement on heart, lung and kidney weights as compared with sham operated controls.** Cardiac, lung, and kidney weights expressed either as absolute values or relative to body weight of rats with compensated and decompensated CHF and their sham controls. Values are means ± SEM. *P<0.05; **P<0.01; ***P<0.001; ****P<0.0001 vs. sham-operated rats. †P<0.05; ††P<0.01; ††††P<0.0001 vs. compensated CHF group.
